# The Role of Novel Approaches and New Findings in the Pharmacology of Tuberculosis Medicines in Improving Treatment Outcomes

**DOI:** 10.1093/cid/ciy710

**Published:** 2018-11-28

**Authors:** Dennis Falzon, Ernesto Jaramillo, Christopher Gilpin, Karin Weyer

**Affiliations:** World Health Organization, Global Tuberculosis Programme, Geneva, Switzerland

**Keywords:** antitubercular agents, pharmacology, drug resistance, microbial, drug monitoring

In its 2017 report on the status of tuberculosis in the world, the World Health Organization (WHO) estimated that tuberculosis causes about 1.7 million deaths each year, making it the single leading infectious cause of death [1]. Standardized regimens with multiple antimicrobials have been the cornerstone of tuberculosis treatment for several decades, and most of the 10 million new tuberculosis cases emerging globally each year can be cured with an affordable 6-month treatment course. A massive roll-out of rifampicin-based first-line tuberculosis regimens has helped save more than 50 million lives since 2000. Nonetheless, the prospects of ending the global tuberculosis epidemic in the coming years are threatened by the spread of drug-resistant strains that complicate treatment. About 600 000 new cases of rifampicin-resistant (RR-TB) or multidrug-resistant tuberculosis (MDR-TB; combined resistance to isoniazid and rifampicin) emerge each year, requiring treatment with multiple medicines and for much longer than with first-line regimens: often 2 years or more. Globally, only about one-half of the MDR/RR-TB cases starting treatment complete it successfully.

A core mandate of WHO is the development of evidence-based policy for public health interventions [2]. The latest WHO treatment guidelines for tuberculosis and MDR/RR-TB were released in 2016 and 2017, using the Grading of Recommendations Assessment, Development and Evaluation (GRADE) approach to assess evidence from the most recently available data on patient outcomes [3–5]. Important uncertainties critical to the safe and effective implementation of drug-resistant tuberculosis care persist, given the limitations of available evidence and the paucity of trials. These pertain to the optimization of dosages and combination of medicines to maximize benefits and limit drug-drug interactions and other harms; adapting treatment for key subpopulations, like children and patients with comorbidities such as human immunodeficiency virus, diabetes, depression, and hepatitis; and interpreting in vitro results from drug susceptibility testing and mycobacterial genome sequencing for clinical decision-making.

WHO convened a technical consultation in Geneva in April 2017 to review the latest data on the pharmacology of those tuberculosis medicines used in MDR-TB regimens [6]. The discussion, both during and subsequent to this expert meeting, considered how the latest knowledge on the pharmacokinetics (PK; how the human body handles a substance), pharmacodynamics (PD; how a substance impacts on an organism or the body), and pharmacogenetics (how genetics influence the metabolism of a substance) of tuberculosis medicines could help answer some of these questions. This special Supplement of *Clinical Infectious Diseases* features a series of papers prompted by the scientific debate in the 2017 WHO expert consultation. The authors explore critical issues of uncertainty in tuberculosis therapeutics, some of them of long-standing. Most of the papers refer to findings from hollow fibre infection models (HFIM), which are increasingly employed to investigate the anti-tuberculosis properties of substances at the preclinical stage to complement the more-conventional tools applied to determine drug/regimen efficacy and safety [7]. HFIM and associated statistical modelling can help identify which PK/PD parameters correlate best with drug efficacy and toxicity.

This Supplement includes a set of articles coordinated by the University Medical Center Groningen that review the literature on PK/PD data for mainstay components of second-line MDR-TB regimens. These systematic reviews formed part of the preparation for the WHO consultation in 2017, and provide a useful update on the PK/PD parameters that are best correlated with the performance of amikacin, linezolid, and cycloserine [8–11].

The other papers analyze data from new or completed studies, often employing novel approaches. In 2 separate articles, Deshpande et al [12] and Srivastava et al [13] use HFIM to compare the performance of linezolid with tedizolid—a related and newer oxazolidinone that is not currently used to treat MDR-TB—and also propose a dosage that would match the sterilizing effect of this new medicine. In 2 other papers, Deshpande et al also use HFIM models to explore bactericidal doses of cycloserine and the doses of gatifloxacin associated with a high probability of cures in pulmonary and meningeal tuberculosis [11, 14]. In 2 other articles, also led by Deshpande, artificial intelligence is employed to enhance the predictive potential of HFIM, in order to find a levofloxacin dose equivalent to moxifloxacin or to identify the determinants of microbiological outcomes in ethionamide-containing regimens [15, 16]. Pasipanodya et al [17] employ artificial intelligence to model the antagonism between the components of an experimental 4-month regimen with rifampicin, pyrazinamide, and gatifloxacin that was also studied in a clinical trial aimed at reducing treatment duration in drug-susceptible tuberculosis [18]. Using data from another study with a similar objective [19], Srivastava et al apply HFIM to model the performance of rifampicin, pyrazinamide, and moxifloxacin at higher doses than those used in the regimen studied in the trial itself [20]. Magombedze et al use data from the same trial of this 4-month moxifloxacin-containing regimen to propose a computational modelling framework as a more precise approach to mapping preclinical results from the HFIM to observed treatment outcomes [21].

The exciting new findings in tuberculosis pharmacology and the novel investigative techniques reported in this Supplement are relevant to the continued development of new medicines, both in pre-clinical and clinical studies. Artificial intelligence could help identify complementary medicinal combinations and dosages to optimize the composition of candidate regimens ahead of the start of trials. In clinical practice, PK/PD knowledge is likely to bring the prospect of precision medicine in public health within closer reach of more patients worldwide, especially if linked to drug discoveries and concurrent advances in diagnostics, therapeutics, and digital technologies. The optimization of drug dosages and treatment schedules for drug-resistant tuberculosis require more scientific debate, along with fresh data of better quality from patients receiving treatment. Therapeutic drug monitoring (TDM) may be relevant to MDR-TB care, due to the narrow therapeutic index and inter-patient variation in PK/PD of fluoroquinolones, injectable agents, linezolid, and other second-line tuberculosis medicines. TDM may become more feasible in resource-limited settings with the addition of advancements in specimen transportation and analytic methods based on mass spectrometry. However, the added value of different TDM strategies on clinical outcomes still needs to be confirmed and quantified.

The conclusions made in the papers of this Supplement do not represent WHO recommendations on the use of the medicines, or an endorsement of experimental medicines, regimens, dosages, or new methods of diagnosis. Nevertheless, in the absence of a more robust evidence base from trials and other studies, these PK/PD updates may help researchers to optimize their efforts and inform health care practitioners to assist them in making better decisions in the clinical management of tuberculosis. This information also serves as a valuable background to the ongoing updates being made by WHO to its MDR/RR-TB treatment policies [22].

## Notes

***Disclaimer.*** All authors are staff members of the World Health Organization (WHO); they alone are responsible for the views expressed in this publication, which do not necessarily represent the decisions or policies of WHO. The designations used and the presentation of the material in this publication do not imply the expression of any opinion whatsoever on the part of WHO concerning the legal status of any country, territory, city, or area; its authorities; or the delimitation of its frontiers or boundaries.

***Supplement sponsorship.*** This supplement is sponsored by the Baylor Institute of Immunology Research of the Baylor Research Institute.

***Potential conflicts of interest.*** All authors: No reported conflicts. All authors have submitted the ICMJE Form for Disclosure of Potential Conflicts of Interest. Conflicts that the editors consider relevant to the content of the manuscript have been disclosed.
